# Emotion expressions and cognitive impairments in the elderly: review of the contactless detection approach

**DOI:** 10.3389/fdgth.2024.1335289

**Published:** 2024-07-08

**Authors:** Di Jiang, Luowei Yan, Florence Mayrand

**Affiliations:** ^1^ Medical Devices Research Centre, National Research Council of Canada, Boucherville, QC, Canada; ^2^ Department of Psychology, McGill University, Montreal, QC, Canada

**Keywords:** cognitive decline, remote health, contactless detection, machine learning, elderly population

## Abstract

The aging population in Canada has been increasing continuously throughout the past decades. Amongst this demographic, around 11% suffer from some form of cognitive decline. While diagnosis through traditional means (i.e., Magnetic Resonance Imagings (MRIs), positron emission tomography (PET) scans, cognitive assessments, etc.) has been successful at detecting this decline, there remains unexplored measures of cognitive health that could reduce stress and cost for the elderly population, including approaches for early detection and preventive methods. Such efforts could additionally contribute to reducing the pressure and stress on the Canadian healthcare system, as well as improve the quality of life of the elderly population. Previous evidence has demonstrated emotional facial expressions being altered in individuals with various cognitive conditions such as dementias, mild cognitive impairment, and geriatric depression. This review highlights the commonalities among these cognitive health conditions, and research behind the contactless assessment methods to monitor the health and cognitive well-being of the elderly population through emotion expression. The contactless detection approach covered by this review includes automated facial expression analysis (AFEA), electroencephalogram (EEG) technologies and heart rate variability (HRV). In conclusion, a discussion of the potentials of the existing technologies and future direction of a novel assessment design through fusion of AFEA, EEG and HRV measures to increase detection of cognitive decline in a contactless and remote manner will be presented.

## 1 Introduction

The cognitive health of the elderly population has grown to be a central issue in our society. Statistics estimated that at least 6.5 million of Americans aged 65 years or over are living with Alzheimer's disease (AD) (1). In Canada, 597,300 individuals were living with dementia in 2020, and this number was projected to reach close to a million by 2030 (2). The demand and reliance on valid and precise diagnostic tools have therefore increased exponentially. Historically, traditional tools such as neuropsychological tests and brain imaging techniques have been the state-of-the-art diagnostic methods and assessment of severity. Although accurate, these techniques involve intense patient participation in the case of tests or intrusive manipulations in the case of MRIs and PET scans. During the same period where AD cases increased, the proportion of elders living in collective dwellings or assisted living facilities, such as a nursing home, a chronic care facility or a residence for seniors, also evolved significantly. The 2011 census indicated that 7.9% of seniors aged 65 or over resided in a collective dwelling, whereas the 2021 census revealed 28% of those aged 80 and above living in such arrangements (3, 4). Specifically with the impact of COVID-19 pandemic, one in every twenty Canadians aged 65 or over were living in these facilities in 2021 (5, 6). Thus, our healthcare systems are facing an unprecedented situation with continuously increasing needs and burdens. The emerging trend of regrouping of patients in the facilities brings on new possibilities regarding the assessments of their disorders and disabilities, such that this environment could serve as both the treatment and the diagnosis method. For instance, remote and contactless tools could easily be integrated into the living installations which AD patients utilize daily. To this end, existing evidence has demonstrated emotional facial expressions being altered in individuals with various cognitive conditions such as dementia, mild cognitive impairment, and geriatric depression. Technologies such as Automated Facial Expressions Analysis (AFEA) and remote photoplethysmography (rPPG) have been shown to provide accurate and reliable measures which can be related to cognitive health and disease progression. Given that these technologies can be added to daily protocols already administered to patients in care facilities via camera recordings, assessment of patients’ health could be completely re-invented such that intrusive methods will be on need-basis and less required, and preliminary diagnosis can occur in community. In this paper, we will review the use of these technologies in the context of various cognitive conditions to enhance the accessibility of treatment and progress tracking for the elderly in a remote and contactless manner.

## 2 Cognitive impairments in the elderly population

### 2.1 Dementia and Alzheimer's disease

Dementia, the most well-known disorder associated with the elderly, is a general term for several diseases, including AD (7). Over 350 Canadians on average were diagnosed with dementia every day in 2022 (2). All these data demonstrate that dementia, with its prevalence, is a non-negligible condition in the health assessment of elders in long-term care facilities. Dementia is understood to affect memory, increase confusion, apathy/depression, and leads to a loss of ability to complete everyday tasks (8). Typically, dementia is assessed through various cognitive and neuropsychological tests such as the Mini-Mental Status Examination (MMSE). Brain scans, such as MRIs and PET scans, can also be used to detect dementia through changes in the brain structure, but these are associated with a high cost and demand extensive resources. While it remains exploratory whether the expression of emotions differs between older adults with dementia and healthy ones, several studies in this space provided promising results. For example, studies looking into facial expression of pain found that participants with dementia expressed more pain in their faces than participants in the control group (9). A more recent study also found that across their participant pool from AD research centers, it appeared that dementia patients facially expressed fewer positive emotions during emotion-eliciting events and instead used more negative expressions (10). In addition, AD patients demonstrated an overall increase in facial expressiveness (11). Similarly, other studies have found altered zygomatic activity (i.e., muscles that control smiling) in patients with dementia while viewing emotion-eliciting images when compared to healthy elderly counterparts (12). The flexibility in emotion expressions was also found to be reduced for AD patients, such that they struggled to amplify positive emotions facially (13). That being said, while these techniques have been used increasingly in the clinical world, no automated assessment of pain through facial expressions has been tested as a valid tool for detecting dementia (14), and little effort has been put into relating facial expression analysis to other physiological measures of dementia. Hence, the automation of facial expression analysis, paired with other measures, would therefore provide an interesting option to both detect dementia, as well as monitor it once it is diagnosed.

### 2.2 Mild cognitive impairment

Dementia is often first diagnosed as mild cognitive impairment (MCI), which makes it one of the first observable conditions and symptoms in one's cognitive decline. MCI is characterized by a limbo state between normal aging and dementia (15). For people with MCI, typical symptoms include memory deficits as well as other reduced cognitive functions that do not hinder or only slightly affect one's instrumental functional abilities. The prevalence of MCI increases with age, with 10.88% of community-dwellers aged 50–59 years and 21.27% of those aged 80 years and above, as indicated by a recent worldwide meta-analysis (16). More importantly, up to 30% of adults who develop MCI will go on to be diagnosed with some form of dementia; typically AD for those who experience memory deficits (17). Early detection of MCI is crucial in reducing one's risk of developing dementia. Traditional detection tools, however, are often targeted towards the impairments found in AD and therefore not very accurate at detecting MCI. In fact, the MMSE, which is one of the most commonly used cognitive tests, can only detect around 18% of MCI cases (18), and it does not provide substantial support for the early detection of dementia in MCI patients (19). Therefore, more tools are necessary to better understand MCI and help early treatment of cognitive decline. It is known that people with cognitive impairments express emotions differently through their faces compared to healthy adults of the same age (20, 21), and a recent effort has been made to use non-invasive, readily available technologies to assess MCI. For example, Fei and colleagues (18, 22) proposed computer vision techniques for the detection of cognitive impairment, including MCI, in the elderly by analyzing facial features. As manual coding of these expressions can be tedious, an automated way of facial expression analysis (i.e., AFEA) could potentially provide an efficient, contactless, and non-intrusive detection tool for MCI and allow for better prevention of dementia.

### 2.3 Depression

Often overlooked, depression is one of the most common symptoms in dementia and MCI patients. The prevalence of depression among elderly individuals tends to vary across investigations due to different experimental designs (23–25). For instance, estimates suggest that depression could affect up to 5% of the elderly population and close to 44% in elders requiring residential health care (26, 27). Despite its prevalence, late onset depression remains underdiagnosed and characterized as a part of normal aging. However, depression has serious impacts on the elderly's cognitive and physical health. Late onset of depression can lead to serious cognitive deficits, often similar to those seen in MCI (28). Research has shown that MCI patients are more likely to develop depression, with a prevalence rate between 16.9% and 55% (29). In fact, half of those who experience depression after the age of 65 and along with cognitive impairment will go on to develop AD or other types of dementia. The comorbidity of depression in patients with dementia can vary between 9 and 68% (30). Hence, depression is both seen as a risk factor for dementia as well as a symptom of the disorder. The cognitive damage due to depression can however be reversed before one progresses into dementia but is too often ignored or undiagnosed. Typical assessments of depression such as the Geriatric Depression Scale (GDS) can be misleading and often wrongly diagnose cognitively impaired adults as depressive (31). New methods of detection are therefore needed, one of which could be the analysis of facial expressions or muscle activity. Facial expression analysis is a common tool used in adults with depression. For example, it has been shown that depressed individuals have a loss of facial muscle tone around the mouth, but higher tone in the brow area, which can be associated with anxiety and anger. Overall, depressed individuals express fewer smiles than healthy adults (32). Depressed individuals also demonstrate decreased activities in the cheek and brow areas upon viewing happy and sad images, compared to non-depressed individuals (33). Correlations between depressive symptoms and end-lip, mouth width, mid-top lip, eye-opening, and mid-eyebrow measures have been found in some studies (34), as well as facial indicators of excess activity in the grief regions of the face, even during joy-inducing stimuli (35). Facial expression analysis is able to identify all these small facial changes during expression of emotions, but it has never been specifically applied to geriatric or late onset depression. Therefore, it would be informative to explore the application of facial expression analysis in the elderly for the prognosis and detection of depression, which in turn could contribute to the monitoring of dementia symptoms and progress in the same population.

Here, we outlined 3 different cognitive and affective conditions present in the elderly population related to cognitive impairment (see Table 1 for summary of reviewed articles). While established diagnostic methods have been successful in identifying these diseases and disorders, recent breakthroughs in contactless technologies show that facial movements during affective states can be used to monitor cognitive decline and the severity of conditions. The implications of such methods are not negligible; remote and contactless assessment would allow for frequent updates on the cognitive health of elders at risk without invasive procedures and at a relatively low cost. Furthermore, the addition of such technologies can easily be integrated into care facilities, which house hundreds of patients in one place. This facilitates the routine assessment of cognitive decline daily, fostering a proactive approach instead of relying on periodic assessments that could lead to significant deteriorations of disorders.

**Table 1:** Summary of previous research on cognitive decline and depression and facial mobility.

Citation	Article Type	Sample	Measures included	Stimuli	Main findings
Kunz et al. (9)	Empirical	42 demented elders 54 healthy elders	Facial action coding system	Pressure stimulation	• Facial responses were significantly increased in demented patients compared to healthy controls. • Facial responses were closely related to the intensity of stimulation for demented patients.
Jiang et al. (10)	Empirical	258 healthy elders 235 AD elders	Vision-based facial expression recognition	Images of object scenes	• AD participants expressed significantly fewer positive emotions, more negative emotions, and higher facial expressiveness. • Facial emotions expressed during the test allowed effective differentiation of AD from healthy participant.
Seidl et al. (11)	Empirical	47 AD patients	Facial action coding system	International affective picture system	• Cognitive decline was related to increased facial expressiveness. • Apathetic symptoms appear to be specifically associated with facial expression in AD.
Burton & Kaszniak (12)	Empirical	13 elders with AD 21 healthy elders	Corrugator and zygomatic electromyography (EMG)	International affective picture system	• Change in zygomatic activity was significantly different between AD and healthy groups, with AD subjects demonstrating an inverted pattern of activity compared to controls.
Henry et al. (13)	Empirical	20 healthy elders 20 AD elders	Expressive emotion behavior coding system	Neutral and amusing video clips	• AD is associated with subtle changes in emotion-expressive behavior. • AD group displayed significantly lower positive affect compared with the control group.
Chen et al. (20)	Empirical	99 patients with frontotemporal dementia (FTD) 45 AD patients 37 healthy controls	Subjective emotional experience	Film clips	• Patients with AD and FTD tended to experience more “mixed emotions” when watching emotionally arousing film clips. • FTD patients reported more positive and negative non-target emotions, whereas AD patients reported more positive non-target emotions.
Pressman et al. (21)	Empirical	36 healthy adults 89 patients with a neurodegenerative disease	Expressive emotion behavior coding system	Three short films	• Participants with FTD tended to express less emotion on their faces than they did through self-report. • Differences within diagnostic subgroups.
Fei et al. (18)	Review	N/A	Facial features analysis; Facial features classification	N/A	• Automatic facial expression analysis has the potential to be used for cognitive impairment detection in the elderly. • May be better to use a local method of facial components alignment, employ static approaches in facial feature extraction and facial feature classification.
Fei et al. (22)	Empirical	61 healthy and cognitively impaired elders	Deep neural network-based emotion analysis system	KDEF dataset; Chinese adults dataset; Chinese elderly people dataset	• The classifier was able to detect the cognitive impairment based on the emotion data from the testing dataset with a detection accuracy of 73.3%.
Katsikitis and Pilowsky (32)	Empirical	21 Parkinson's disease patients 20 depressed patients 12 healthy adults	Facial Expression Measurement program	12 humorous cartoons	• Depressed patients shown smaller mid-eyebrow measures compared to the control group. • Depressed and parkinsonian group had significantly less smiles.
Gehricke and Shapiro (33)	Empirical	11 depressed patients 11 healthy adults	Facial EMG	Imagery situations	• Facial muscle activity over the brow and cheek region was reduced in depressed compared to healthy patients during happy and sad imagery. • Lack of social context differences in frowning may suggest social disengagement and an inhibition of sad facial expression.
Stolicyn et al. (34)	Empirical	48 depressed participants	Facial action coding system	Delayed match to sample task; Rapid detection task; Affective distractions	• Symptomatic participants were characterised by less intense mouth and eyelid movements. • Classification accuracy using cross-validation (within-study replication) reached 79%.
Greden et al. (35)	Empirical	29 healthy controls 37 depressed adults	Facial EMG	Imagery situations	• Patients with endogenous depression had EMG levels that differentiated them from healthy subjects. • Depressed participants had significantly greater activity in corrugator happy and corrugator sad imagery trials.

## 3 Automated facial expression analysis, EEG and rPPG in emotion recognition

In recent years, contactless detection for facial expression analysis and emotion recognition has become a growing field, with more interest in its applications in the medical health domain. Today, various automated methods for emotion assessment have been developed to increase the accuracy of emotions through different means. Among them, Facial Expression Analysis, EEG, and heart rate monitoring have been emerging as viable ways to understand human emotions. Both Facial Expression Analysis and heart rate monitoring have been made available through contactless and remote means such as AFEAs and rPPG, respectively. In this section, we will present a summary of these technologies together with their respective accuracy and usability, as well as express the need for joint usage of these methods in emotion detection.

### 3.1 Facial action coding system and automated facial expression analysis (AFEA)

The Facial Action Coding System (FACS) is a taxonomy system to identify and classify facial movements during expressions of emotions (36). FACS has been used by psychologists for decades and has recently been applied in animations (37, 38). To classify certain facial expressions, FACS uses Action Units (AUs) to pair together different movements by facial muscles (39). A total of 46 main action units makes up FACS, through which 7 emotions can be detected: happiness/joy, sadness, surprise, fear, anger, disgust, and contempt. Traditionally, FACS required coding of AUs by human coders. The training required to become a certified FACS coder is lengthy, with over hundreds of hours spent coding (40). In the last decade, amazing efforts have been made to automate FACS coding to speed up the process and alleviate human efforts. Through deep learning networks, algorithms have been able to successfully track facial movements and AUs, and subsequent emotion classifications (40–43). Analyses on the accuracy of these algorithms have varied, with some reaching nearly 90% accuracy while others fail to reach 50% accuracy (44–46). For this reason, there are many different algorithms available that use different methods to develop their AFEA using FACS. Certain technology companies have created “ready-to-use” platforms that can serve multiple usage and provide AFEA to a wide range of professionals. Such products, like iMotions's Affectiva and Noldus’ FaceReader, allow for AFEA to occur with video recordings and without the input of human coders. These platforms all operate under the same rules and mostly use similar algorithms to classify emotion expressions (45). When using these algorithms, the choice of camera hardware to record the data is important, as the resolution will factor into the facial feature detection accuracy. Studies show that cameras that have stable framerates, auto-focus, and allow access to aperture, brightness, and white balance settings offer the best results (47).^1^ The Microsoft Kinect RGB-D camera was also found to accurately locate facial features with high resolution (48–51). However, detailed specifications on the appropriate hardware requirements have not been well established.

From a software algorithm perspective, most deep learning networks utilize the Viola-Jones algorithm to detect the presence of faces within an image or video. The Viola-Jones algorithm works by first selecting Haar-like features in images (52). It then creates an integral image and goes through a machine learning algorithm that identifies the best features to detect a face by creating classifiers. Based on which classifiers work the best on training datasets with faces, the best performing ones are kept and then used to discard non-faces in images through a cascade of classifiers. In the last stage, an image is finally classified as a human face. Upon successful face identification, platforms like FaceReader make a 3D model of the face using the Active Appearance Method (AAM) (53). The AAM can locate 500 points on the face and also analyze texture. Based on the location of these points, the AAM can classify facial expressions through the training of the algorithm with over 10,000 images of faces. Once an expression is classified, these platforms can assess the valence and arousal of the expression as well as the intensity of all AUs involved during the expression (53).

Such models and platforms, while having clear advantages and benefits of not needing any pre-programming, require commercial licenses that involve regular payments. In addition, studies have demonstrated their limited suitability to applications. Because they are already pre-trained with some generic datasets of face images, some biases were observed in specific populations (54). While somewhat accurate at detecting AUs in the general adult Caucasian population, some research has found that the accuracy of these models drops significantly when applied to other ethnicities and different age groups [(44, 54, 55), but see (56) for new technology addressing AI bias of skin tone]. Therefore, their usage cannot be applied universally.

Nonetheless, the core foundations of these platforms remain unbiased prior to the training of the algorithms. Independent implementation of a similar platform can be done by utilizing open access deep learning networks. Through the training of the network, a platform could hypothetically be applied to any specific group and obtain accurate readings of facial expression. The challenge, however, consistent with those of most AI/ML algorithms, is the need for large-volume, diverse, and well-representing datasets, which are known to be rare and limited (57). To successfully train an AFEA, thousands of images need to be presented in training to develop highly reliable classifiers. Without such training, the algorithm's accuracy will drop significantly, if not be nonexistent. Furthermore, to apply an AFEA to the aging population to detect conditions such as dementia and Parkinson's disease, an extensive collection of images of elderly people's faces would be necessary to train the algorithm. However, because these clinical populations are less prevalent relative to healthy populations, very few datasets are available (58). Among these few are the University of Regina's Pain in Severe Dementia dataset and the UNBC-McMaster Shoulder Pain Expression Archive dataset. Other larger datasets such as the FACES dataset contain a subcategory with older adults but cannot be used on its own (57). Despite the individual limitations, these smaller datasets could potentially be grouped together to train an algorithm to work on the elderly population. Interesting alternatives were explored by researchers responding to the scarcity of available datasets. For example, online videos, such as YouTube videos, with people involved with Parkinson's disease were used to train the AFEA to recognize patterns of the disease without having to develop their own dataset (59). This proved to be a promising training technique, with an accuracy of over 82% for the detection of Parkinson's disease reported. Such a method could be used on all populations that are underrepresented in large datasets (59). Therefore, the biases seen in most algorithms can be minimized through re-training using various databases and available images/videos.

### 3.2 EEG and emotion recognition

In parallel to the externally observable and accessible factors of the facial mobility approach to cognitive assessment, measurement and understanding of patients’ internal brain activity using EEG data has been considered often as a reference information for clinical evaluation. For this purpose, EEG has been extensively studied in different populations exhibiting cognitive decline as well as in demented patients (e.g., AD patients; see Table 2 for summary of reviewed articles). As a result, there has been a growing consensus within the scientific community regarding the overall significance of this approach.

**Table 2:** Summary of previous research on cognitive decline and EEG.

Citation	Article type	Sample	Measures included	Stimuli	Main findings
Meghdadi et al. (60)	Empirical	26 AD patients 53 MCI patients 246 healthy controls	Resting state EEG	N/A	• Increases in both spectral power and coherence at slower frequencies in ADs and MCIs. • Decreases in spectral power at slower frequencies in HCs with advancing age. • Decreases in the spectral power and coherence at Alpha frequency in ADs, but not in MCIs. • Theta-to-alpha ratio demonstrated the largest and most significant differences between ADs and HCs.
Özbek et al. (61)	Empirical	47 early-onset AD patients (EOAD) 51 late-onset AD patients (LOAD) 49 young healthy controls 51 old healthy controls	Resting state EEG	N/A	• Increases in slow frequency bands and decreases in fast frequency bands in EOADs. • Frontal theta-to-alpha ratio best discriminated between EOADs and young HCs. • More widespread and severe electrophysiological abnormalities in EOADs than LOADs and HCs.
Gaubert et al. (62)	Empirical	314 preclinical AD adults	Resting state EEG	N/A	• Increases in high frequency oscillations and decreases in low frequency oscillations in frontocentral regions. • Different EEG patterns modulated by the degree of amyloid burden.
Palmiero et al. (63)	Mini-review	N/A	Various	N/A	• Contradictory and mixed results. • Increases in left prefrontal EEG activity for approach-related and positive emotions. • Increases in right EEG prefrontal activity for withdrawal-related and negative emotions.
Kisley et al. (64)	Empirical	51 healthy adults	Tasked-evoked EEG	Emotion-eliciting images	• Overall larger LPP amplitudes elicited by negative than by positive images. • Linear decline of LPP amplitudes with advancing age towards negative images • Responses towards positive images remained age invariant
Tsolaki et al. (65)	Empirical	11 young adults 11 elderly adults	Tasked-evoked EEG	Photographs of fear and anger facial expressions	• Larger amplitudes of the N170 early component in elderly adults than in young adults. • Less differentiation of N170 topographic maps between the two negative stimuli in elderly than in young adults. • More differentiation of topographic maps between the age groups in ‘anger’ than in ‘fear’.
Güntekin et al. (66)	Empirical	30 healthy controls 30 AD patients	Tasked-evoked EEG	Photographs of angry, happy, and neutral facial expressions	• HCs: increased Theta power towards angry expressions, and increased right hemispheric alpha power. • ADs decreased Theta power towards angry expressions, and decreased right hemispheric alpha power. • Increases in alpha power towards angry than towards neutral expressions.

In resting-state EEG recordings, AD and MCI patients showed an increased spectral power and functional connectivity in the theta and delta bands, which are the slower frequencies of the spectrum (60). Interestingly, participants in the control group showed a decreased spectral power in these bands with advancing age, thus indicating an inverse aging pattern in the AD and MCI groups. AD participants also showed a decreased spectral power and functional connectivity in the alpha band normally observed in healthy aging. Meghdadi et al. (60) also reported that a [theta/alpha] ratio was very good at discriminating AD from MCI and controls, as exhibiting higher values was associated with increasing cognitive impairment and disease progression. Similarly, early-onset AD patients exhibited higher spectral power in the lower frequencies as well as lower spectral power in higher frequencies when compared to age-matched healthy individuals (61). High accuracy was obtained in discriminating the groups by computing a [alpha/theta] ratio, especially when measured in the frontal regions. Moreover, several factors related to different etiologies can explain the clinical symptoms of AD, such as the level of neurodegeneration and the accumulation of the amyloid-beta peptide. Separating groups based on these two variables, Gaubert et al. (62) reported that the most notable effects of neurodegeneration on EEG measures were concentrated in the frontocentral regions. This was marked by a rise in high-frequency oscillations (i.e., higher beta and gamma power), along with a decline in low-frequency oscillations (i.e., lower delta power). In addition, when measuring changes in EEG features after taking amyloid burden into account, the authors reported heterogeneity in participants where the extent of amyloid-beta accumulations can lead to differential spectral power profiles.

Numerous studies have also used EEG to explore brain activity related to emotional processing (67–69). For instance, greater activity in the left prefrontal cortex was found to be associated with approach- related positive emotions, while greater activity in the right prefrontal cortex was associated with withdrawal-related negative emotions (63). In a study by Kisley et al. (64), the researchers examined the late positive potentials (LPP) [i.e., event-related-potentials (ERPs) reflecting enhanced attention to emotional stimuli] in adults ranging from 18 to 81 years old. They found that the LPP amplitudes towards negative images declined linearly with age but remained consistent across ages for positive images. Moreover, Tsolaki et al. (65), reported that healthy older adults demonstrated larger N170 amplitudes than healthy young adults when viewing facial images displaying anger and fear expressions. Despite these prolific findings, few studies have delved into the impairment of facial recognition in elders with dementia using EEG. One recent study reported that AD patients were shown to have lower theta power than healthy controls when perceiving angry facial expressions (66), suggesting the possible implication of EEG for assessing emotional processing in patients with neurocognitive disorders.

Overall, there is an agreement that there is a decrease in EEG activity in cognitive decline, with higher relative spectral power in the slower frequencies when compared to cognitively unimpaired participants. However, the accessibility and the applicability of EEG sensor devices limit the usage of EEG signals for cognitive skill evaluation, especially for the cognitive decline measures for the aging population. Thus, using findings in spectral power across different frequency bands to validate features from facial mobility could help in identifying which features from the automated facial expression analysis are relevant in the remote assessment of cognitive decline in the elderly population.

### 3.3 Heart rate and emotion recognition

In recent years, a strong effort has been made to develop contactless technologies to monitor health through physiological measures. Among them, rPPG has been used increasingly in the medical field to assess heart rate (50) and further introduced in emotion analysis (see Table 3 for summary of reviewed articles). Heart rate variability (HRV) and heart rate (HR) as in beats per minute (bpm), while typically measured through electrocardiogram, have successfully been studied using PPG technologies (70, 74). Indeed, it is now believed that HRV can serve as a basis for recognizing emotions, detect stress and overall identify changes in the Autonomic Nervous System (71, 75). According to a systematic review conducted by Cheng et al. (72), patients with dementia or neurocognitive disorders generally exhibit lower resting HRV indices compared to healthy controls. However, after distinguishing between different types of disorders, significant differences in HRV values are observed only in patients with Dementia with Lewy Bodies and MCI. On the contrary, there are no significant differences between patients with AD, Vascular Dementia, and Frontotemporal Dementia and the healthy controls. Furthermore, rPPG has been used in the study of pain and detection of engagement (14, 51). Because physiological, cognitive, and affective events can cause fluctuations in HRV, rPPG can effectively isolate these changes and attribute them to various states (50). Software platforms, such as the FaceReader, have been used in multiple research studies as the heart rate monitoring tools. Great results have been found using this technology, and it remains the most accessible and well-developed rPPG on the market (14). However, accuracy of the physiological monitoring with these software tools remains to be further validated, and their individual usage as a cognitive assessment tool also requires further testing (73).

**Table 3:** Summary of previous research on cognitive decline and heart rate.

Citation	Article type	Sample	Measures included	Stimuli	Main findings
Lu et al. (70)	Empirical	42 healthy adults	Resting electrocardiogram (ECG); Resting earlobe PPG	N/A	• Correlations in the temporal and frequency domains and in nonlinear dynamic analyses between HRV indices derived from PPG and ECG. • PPG can be a practical alternative to ECG for HRV analysis.
Benezeth et al. (71)	Empirical	16 healthy adults	Camera-based rPPG; Contact sensor-based PPG	Video datasets of participants watching videos eliciting fear or anxiety	• High agreement between the HRV analyses derived from the camera data and contact sensor. • Strong correlation between the remote HRV feature and different emotional states.
Cheng et al. (72)	Systematic review and meta-analysis	Dementia patients healthy controls	N/A	N/A	• Lower resting HRV in dementia patients for parasympathetic functions and total variability compared to HCs. • Lower HRV in patients with MCIs and with Dementia with Lewy Bodies compared to HCs. • Lower HRV in patients with Dementia with Lewy Bodies compared to ADs.
Castillo et al. (14)	Empirical	2 elders with dementia 2 healthy elders	rPPG; Manual FACS coding; Video Magnification (VM) algorithm	Video datasets of pain patients	• Correlation between automated FaceReader™ HR estimates and the optimized VM algorithm in baseline and pain conditions. • Correlation between non-verbal automated FaceReader™ pain scores and manual FACS coding. • rPPG can be useful for the automated estimations of HR values and non-verbal pain scores.
Benedetto et al. (73)	Empirical	24 healthy adults	rPPG; ECG	Stress test (i.e., Go/No-Go task)	• Poor accuracy in FaceReader™ rPPG compared to ECG, especially for lower and higher heart rates. • Lack of studies validating consumer devices and more assessment should be conducted.

### 3.4 Data fusion of heart rate, EEG and AFEA

In an effort to increase accuracy in emotion analysis, some studies have paired rPPG measures with AFEA to establish meaningful correlations between the facial expressions and the physiological measures of emotions (49, 70; see Table 4 for summary of reviewed articles). Interestingly, this pairing allows for both strong (i.e., surprise, fear, joy, etc.) and subtle (stress, contempt, etc.) affective states to be identified. rPPG relies on the discrete changes in heart rate to identify these subtle emotions, while AFEA is successful at differentiating between strong emotions that elicit similar variations in heart rate (77). This fusion of measures ensures that micro-expressions, notorious for escaping AFEAs due to their lack of intensity, are still detectable and accounted for (78).

**Table 4:** Summary of previous research on data fusion of AFEA, rPPG, EEG, and other measures.

Citation	Article type	Sample	Measures included	Stimuli	Main findings
Monkaresi et al. (51)	Empirical	23 healthy adults	Heart Rate (HR); Facial-Feature Based Detection of Engagement	Essay writing	• Accuracy of 75.8% when detecting engagement with pairing facial features and HR in live recordings. • Results were best when data fusion was used, over just facial-feature based detection.
Pham and Wang (76)	Empirical	24 healthy adults	Implicit photoplethysmography (PPG); Facial expression analysis (FEA)	Video advertisements clips	• AttentiveVideo achieved good accuracy (73.59%) on a wide range of emotional measures. • FEA works better for strong emotions (e.g., joy and anger), the PPG channel is more informative for subtle responses or emotions.
McDuff (77)	Doctoral Thesis	N/A	Automated facial expression analysis; Remote measurement of physiology	N/A	• There are clear trends within the physiological responses of individuals and the affect of the content they are watching. • Occurrences of positive valence expressions were predictors of increased preference toward presented stimuli.
Lei et al. (78)	Empirical	Healthy adults	Automated facial expression analysis (iMotions); Galvanic skin response (GSR); Heart Rate (HR)	Emotional videos	• Higher correlation between emotion and GSR compared to emotion and heart rate. • Within a participant, there was no distinct pattern found with the levels of the three parameters measured.
Nagasawa et al. (79)	Empirical	35 health adults	Electroencephalograph (EEG); Automated Facial Expression Analysis; Heart Rate (HR)	FilmStim database	• Noncontact measurement features can be estimated more accurately than EEG extracted features. • Compared to using only facial expressions, combining multiple physiological signals like HR enabled more accurate estimations.
Sun et al. (80)	Empirical	12 healthy adults	Functional near-infrared spectroscopy (fNIRS); Electroencephalograph (EEG); Automated Facial Expression Analysis	Emotional videos	• Results reveal a strong correlation between spontaneous facial affective expressions and the emotional valence. • The affective states were estimated by the fNIRS + EEG brain activity measurements. • Joint utilization of facial expression and wearable neuroimaging for improved emotional analysis.
Koelstra and Patras (81)	Empirical	24 healthy participants	Electroencephalograph (EEG); Automated facial expression analysis	Film clips	• A feature-level fusion approach is demonstrated to improve upon single modality results. • The differences are small and the number of samples too limited to provide a definite answer on the benefits of fusion.

While heart rate monitoring and EEG measures have both individually been paired with automated facial expression analysis establishing correlations, only one recent study investigated employing a multimodal method to increase the accuracy in evaluating emotional states. Nagasawa et al. (79), presented participants with emotion-eliciting videos and obtained their facial recordings as well as EEG signals. Facial recordings were later analyzed to extract physiological responses (i.e., facial expressions, HR, and changes in pupil diameter). After performing an estimation on all data, researchers correlated them with participants’ subjective ratings. Results showed a stronger correlation between the estimated arousal signal derived from physiological responses and subjective ratings, compared to those derived from EEG signals, and a similar trend was observed for valence. Therefore, it appears that a multimodal measurement does improve the accuracy of estimating emotions to some extent.

Establishing links between these three measures is imperative in the study of emotions, mostly because they all serve different purposes. If one of these factors can be measured, inferences can be made about the state of the other two. EEG signals can establish the reference value of emotions one is feeling, even if they are not facially expressed (i.e., sadness while smiling). HRV and HR signals are especially indicative of subtle emotions, as seen in prior literature (74, 76). AFEA performs well when detecting strong emotions that are visible through facial movements. Hence, they are all necessary in their own rights in emotion detection. EEG requires extensive equipment and professional guidance to be accurately performed, which is not feasible in the context of remote and contactless emotion analysis, thus only rPPG and AFEA can be used. Considering the established correlations between heart rate variability and brain signals, EEG might not be indispensable in this context. True emotions can be attributed based on heart rate monitoring and therefore replacing EEG in emotion detection. In the case of establishing these correlations with contactless technologies, one would need to conduct a joint study to ensure that past correlations that have been found in EEG signals, heart rate monitoring and facial expression analysis still hold true in contactless technologies (rPPG and AFEA).

In the case of AFEA and EEG specifically, several studies have shown that EEG data can be used to classify different emotion categories processed by participants. Wang et al. (82) reported that the power spectrum was the best EEG analysis method to classify the emotional valence (i.e., positive, or negative) of the stimuli presented. In this study, higher frequency bands (i.e., beta and gamma frequency bands) were shown to have increased robustness at discriminating the valence component of emotions. In addition, a classification algorithm using the spectral power on different channels was able to classify both the emotional valence and arousal of the emotion processed by participants with a high accuracy (83). These findings indicate that using a relatively basic analysis method of the EEG signal such as spectral power can provide insight into certain components of the emotions being processed by participants, such as arousal and valence. For instance, the combination of EEG features and spontaneous facial expression leads to high accuracy in emotional valence classification (80). This suggests a potential relationship between EEG activity and facial expressions regarding emotional processing, and each of these modalities can offer unique insights. Furthermore, when comparing EEG and facial features on different dimensions of emotional processing, it has been shown that both modalities perform equally at classifying arousal, but that EEG was better at classifying the valence of the emotional stimuli (81). Thus, it appears that facial features can inform about the integrity of emotional processing with an accuracy as good as EEG. This increases the confidence in using automated facial expression analysis to assess emotional processing and as it was discussed in the first section, it is possible to extend this to the assessment of cognitive integrity.

## 4 Discussion

In the present review, we have highlighted three inter-connected cognitive conditions across the elderly population that lack easily accessible, non-invasive detection and progression methods: dementia, MCI, and geriatric depression. More specifically, facial expressions and emotional responses, clear indicators of cognitive decline, have yet to be utilized in the clinical assessment of these conditions. The findings reported here show that there is a link to be made between facial expression features and cognition by assessing emotional processing. We therefore put forth the use of facial expression analysis, augmented by physiological measurements, within the established assessment of these conditions to enhance the accessibility of treatment and progress tracking for the elderly.

As stated in the earlier sections of this review, the current state of the methods used in this clinical area leads to the conclusion that the remote assessment of automated facial expression analysis through the presentation of emotionally charged stimuli with the purpose of assessing cognitive integrity should be further investigated. Given that we can observe changes in muscle tone and activity through passive viewing of such images, the monetary and time cost of cognitive evaluation could be significantly reduced. Although promising, the links between facial expressions during emotional states and cognitive health needs to be validated across various conditions, particularly for the aging population where various levels of cognitive deficits might be present. Hence, the validation of this assessment with the use of EEG analyses will provide increased confidence in the development of robust methods of remote cognitive decline detection.

The potential avenues that stem from these technological developments are not negligible. If facial expression analysis is validated as a viable tool to as an indicator of the progression of cognitive health, the necessary technologies could be implemented within the care centers (i.e.,: a nursing home, a chronic care facility or a residence for seniors) where the elderly are living. The monitoring of their conditions can therefore occur daily via cameras, for example, placed in common living areas and information can be automatically extracted and analyzed by their healthcare provider. This significantly reduces the need for mobility for the elderly to access continuous healthcare. The movement towards automated and in-house health monitoring is already underway, with many products now available to connect individuals to their provider in the comfort of their homes [see Philip et al. (84) for a review of the current technologies for at-home health monitoring for the elderly].

Overall, the combined use of these technologies in emotion recognition provides an increase in accuracy, for both strong and subtle emotions and states. Through such methods, one could potentially obtain true affective states while analyzing the expressed facial movements in order to better understand cognition and emotion processing. These technologies would allow us to move health and medical monitoring into a completely automated phase, in which minimal professional input is needed while profiting the patients. Future work should focus on establishing valid and reliable links between emotional facial expressions and brain activity as well as testing the acceptance of such technologies in the elderly population.

## Footnotes

^1^The Logitech HD Pro webcam C920 seems to obtain the best results amongst webcams (45).
